# Protein-Bound Uremic Toxins and Cognitive Function: A Cross-Sectional Study in a Community-Based Cohort

**DOI:** 10.3390/toxins18100422

**Published:** 2026-09-30

**Authors:** Suguru Yamamoto, Takahiro Tanaka, Nobutaka Kitamura, Miho Kawachi, Akio Yokoseki, Minako Wakasugi, Osamu Onodera, Naoto Endo, Masakazu Wada, Kenji Sato, Kaori Kikuchi, Ayano Konagai, Shin Goto, Ichiei Narita

**Affiliations:** 1 Division of Clinical Nephrology and Rheumatology, Kidney Research Center, Graduate School of Medicine, Dentistry and Health Sciences, Niigata University, Niigata 951-8510, Japan; mmcha2mimicxx691@icloud.com (M.K.); gotos@med.niigata-u.ac.jp (S.G.); naritai@icloud.com (I.N.); 2 Clinical and Translational Research Center, Niigata University Medical and Dental Hospital, Niigata 951-8510, Japan; ta-tnk@med.niigata-u.ac.jp (T.T.); nktmr@m12.alpha-net.ne.jp (N.K.); 3 Department of Inter-Organ Communication Research, Graduate School of Medical and Dental Sciences, Niigata University, Niigata 951-8510, Japan; yokosekiakio@gmail.com (A.Y.); minakowa@med.niigata-u.ac.jp (M.W.); onodera@bri.niigata-u.ac.jp (O.O.); nendo120@gmail.com (N.E.); 4 Sado General Hospital, Niigata 952-1209, Japan; wada@sado-hp.jp (M.W.); kenko@js2.so-net.ne.jp (K.S.); 5 Research and Development Division, Kureha Corporation, Tokyo 103-8552, Japan; fujinami@kureha.co.jp (K.K.); konagai@kureha.co.jp (A.K.)

**Keywords:** chronic kidney disease, cognitive impairment, protein-bound uremic toxins, indoxyl sulfate, Mini-Mental State Examination

## Abstract

Cognitive dysfunction is increasingly recognized as a significant complication of chronic kidney disease (CKD), but the clinical relevance of protein-bound uremic toxins (PBUTs) to cognition remains insufficiently characterized. This cross-sectional analysis included 1217 community-dwelling individuals. Cognitive function was assessed using the Mini-Mental State Examination (MMSE), with scores < 28 indicating possible mild cognitive impairment (MCI). Serum concentrations of five PBUTs—indoxyl sulfate, p-cresyl sulfate, phenyl sulfate, indoleacetic acid, and hippuric acid—were measured, and a composite PBUT score was derived using principal component analysis. Multivariable logistic regression was used to examine the associations of MCI with the PBUT score and with each PBUT. Serum PBUT concentrations increased with advancing CKD stage. The mean MMSE score was 26.7 ± 3.4, and scores progressively declined with advancing CKD stage. A higher PBUT score was independently associated with MCI (coefficient, 0.290 ± 0.077; odds ratio, 1.337; 95% confidence interval, 1.151–1.554; *p* < 0.001). In conclusion, a greater PBUT burden was independently associated with cognitive impairment. These findings identify PBUT burden as a potential correlate of cognitive impairment in CKD. Prospective studies are needed to clarify its temporal relationship with cognitive decline and to assess whether it provides predictive value beyond established measures of kidney function.

## 1. Introduction

Progression of chronic kidney disease (CKD) increases the risk of end-stage kidney disease (ESKD), the need for kidney replacement therapy, and the development of systemic complications [1]. These complications adversely affect survival, activities of daily living (ADL), and quality of life (QOL) [2,3]. In addition to preserving kidney function through appropriate treatment, early identification of CKD-related systemic complications is essential to prevent or delay their progression.

Cognitive impairment is a particularly important systemic complication of CKD [4,5,6]. A previous meta-analysis reported that cognitive impairment affected approximately 32% of patients with nondialysis CKD [7]. Cognitive impairment arising early in the course of CKD can reduce ADL and complicate disease self-management, including management after dialysis initiation, underscoring the importance of early detection and intervention. Although its pathophysiology remains incompletely understood, both traditional and CKD-specific factors appear to contribute [5].

The serum concentrations of uremic toxins increase with CKD progression [8,9]. Accumulation of these toxins contributes to the deterioration of kidney function and to the onset and progression of CKD-related disorders [8,9]. Among them, protein-bound uremic toxins (PBUTs) including indoxyl sulfate (IS) and p-cresyl sulfate (PCS) are particularly toxic, as demonstrated in basic research, and conventional dialysis or hemodiafiltration remove them inefficiently because of their high protein-binding properties [10]. For example, basic and clinical studies have shown that IS is associated with atherosclerotic stiffness and vascular calcification in large vessels [11,12]. Furthermore, experimental studies have suggested that IS accumulates in brain parenchyma [13] and affects neuronal cells [14,15].

Given these findings, PBUTs may contribute to cognitive dysfunction in patients with CKD. However, clinical studies investigating this association, especially among patients with nondialysis CKD, remain limited. In this study, we conducted a cross-sectional analysis of a community-based cohort to examine the association between uremic toxins, particularly PBUTs, and cognitive function.

## 2. Results

### 2.1. Study Samples

The analysis included 1217 participants who underwent cognitive function assessment using the Mini-Mental State Examination (MMSE). Table 1 presents the participants’ characteristics. Male participants accounted for 52.7% of the study population, and the mean age was 65.5 ± 12.11 years. The mean body mass index (BMI) was 23.9 ± 4.0 kg/m^2^, and the mean systolic blood pressure was 137.5 ± 29.9 mmHg. Diabetes mellitus was present in 34.1% of participants, and histories of myocardial infarction and cerebrovascular diseases were observed in 5.0% and 12.1% of participants, respectively. The mean estimated glomerular filtration rates based on creatinine (GFRcre) and cystatin C (GFRcys) were 79.6 ± 79.4 mL/min/1.73 m^2^ and 60.5 ± 26.5 mL/min/1.73 m^2^, respectively. The mean serum β_2_-microglobulin (β2-m) level was 4.0 ± 6.1 mg/L.

### 2.2. Cognitive Function and Kidney Function

**Figure 1:**
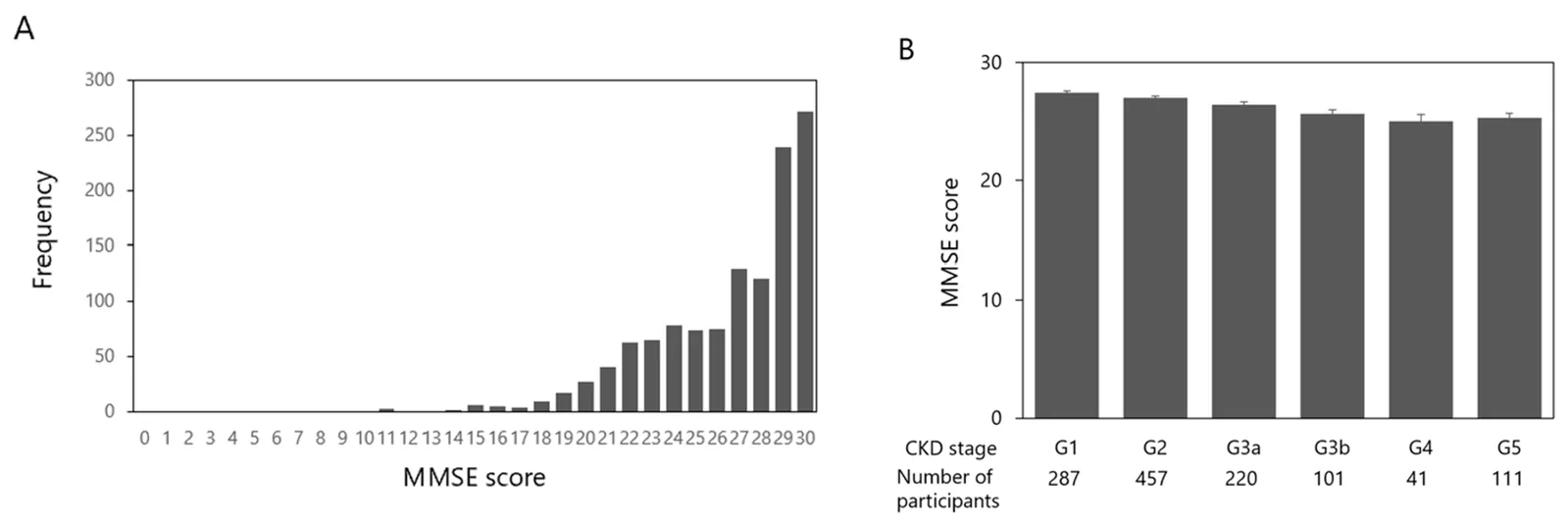
Cognitive function in the study population. (**A**). Distribution of MMSE in the participants. (**B**). Severity of cognitive dysfunction with CKD categories, including CKD G1 (GFRcre 90 mL/min/1.73 m^2^), G2 (GFRcre 60–89 mL/min/1.73 m^2^), G3a (GFRcre 45–59 mL/min/1.73 m^2^), G3b (GFRcre 30–44 mL/min/1.73 m^2^), G4 (GFRcre 15–29 mL/min/1.73 m^2^), and G5 (GFRcre < 15 mL/min/1.73 m^2^). GFRcre, glomerular filtration rate estimation using standardized serum creatinine; MMSE, Mini-Mental State Examination.

The mean MMSE score among all participants was 26.7 ± 3.4. Figure 1A shows the distribution of MMSE scores across the study population. Among all participants, 48.2% had an MMSE score of <28, suggesting mild cognitive impairment (MCI). Mean MMSE scores for CKD stages G1, G2, G3a, G3b, G4, and G5 were 27.4 ± 2.92, 27.0 ± 3.08, 26.4 ± 3.54, 25.6 ± 3.44, 25.0 ± 3.91, and 25.3 ± 4.02, respectively, and showed a decreasing trend with declining kidney function (Figure 1B).

### 2.3. Protein-Bound Uremic Toxins and Kidney Function

Mean serum levels of PBUTs were 0.358 ± 0.894 mg/dL for IS, 0.665 ± 1.016 mg/dL for PCS, 0.036 ± 0.034 mg/dL for indoleacetic acid (IAA), 0.251 ± 0.574 mg/dL for phenyl sulfate (PhS), and 0.313 ± 1.130 mg/dL for hippuric acid (HA). Each toxin level increased with advancing CKD stage (Figure S1).

### 2.4. Cognitive Function and Protein-Bound Uremic Toxins

For the primary analysis, participants with MMSE scores < 28 were classified as having possible MCI on the basis of previous reports [16,17]. Compared with participants who had MMSE scores ≥ 28, those with scores < 28 were older, more likely to be male, and had a lower BMI and a higher prevalence of previous myocardial infarction and cerebrovascular disease (Table 1). They also tended to have lower GFR values and higher serum β2-m levels (Table 1).

**Figure 2:**
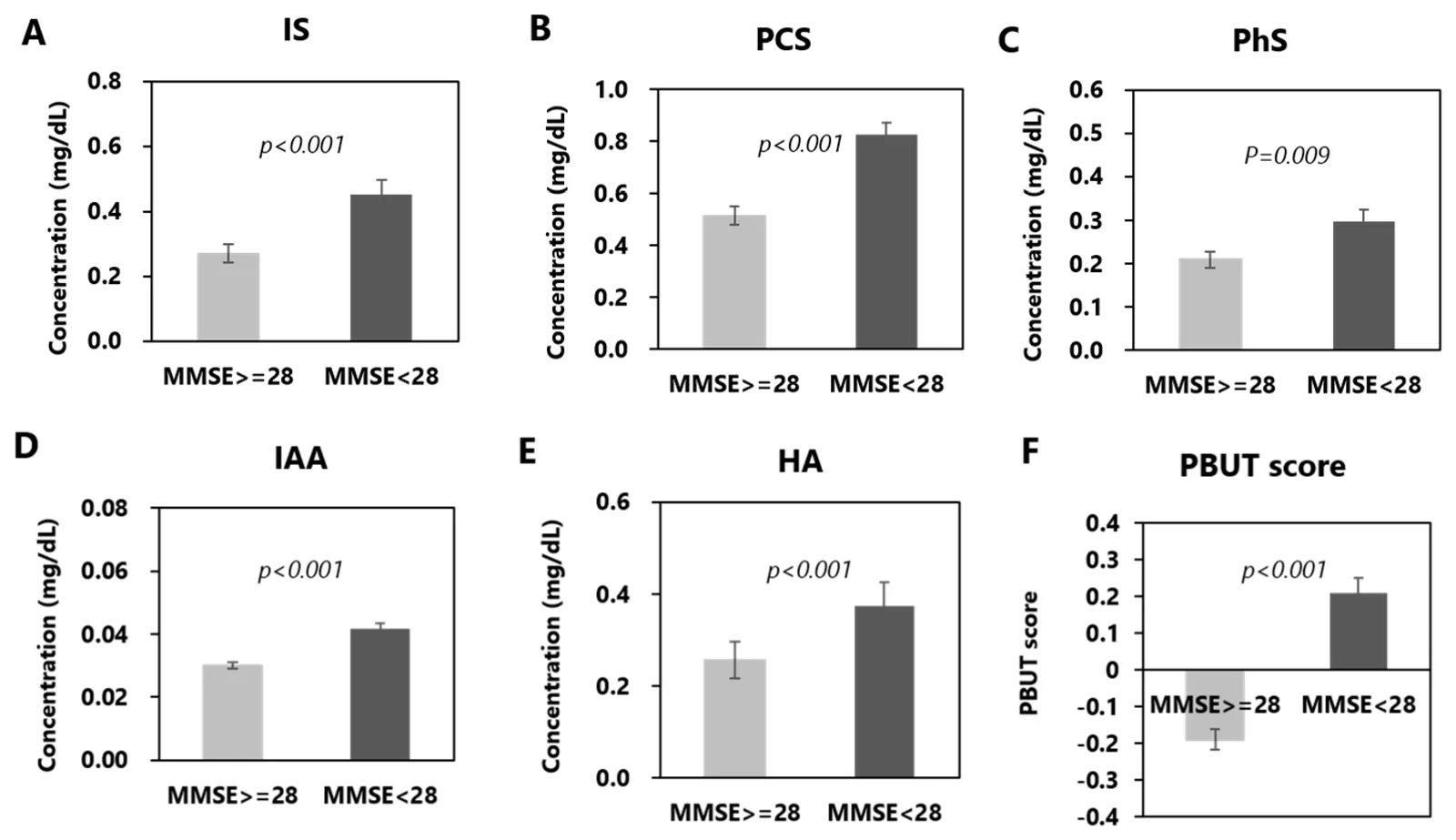
Protein-bound uremic toxin (PBUT) and mild cognitive impairment. Mild cognitive impairment was determined with an MMSE score of <28 points. Serum levels of PBUTs, including (**A**) indoxyl sulfate (IS), (**B**) p-cresyl sulfate (PCS), (**C**) indole acetic acid (IAA), (**D**) phenyl sulfate (PhS), (**E**) hippuric acid (HA), and (**F**) PBUT score, were shown in the participants with or without mild cognitive dysfunction. Data are shown as the mean ± standard deviation (error bars), and *p*-values were calculated using the nonparametric Mann–Whitney U test.

Serum levels of IS, PCS, IAA, and PhS were significantly higher in individuals with MMSE scores < 28 than in those with scores of ≥28 (Figure 2A–E).

Because PBUTs may contribute to cognitive impairment through multiple pathways, a composite PBUT score was derived from standardized concentrations of IS, PCS, IAA, PhS, and HA using principal component analysis. The corresponding component loadings were 1.000, 0.684, 0.634, 0.781, and 0.470, respectively (Table S1). The first principal component (PC1) explained 60.7% of the total variance and had an eigenvalue of approximately 3.04. The scree plot showed a clear inflection between PC1 and PC2, supporting retention of PC1 as the PBUT score (Figure S2). Participants with MCI had higher PBUT scores than those without MCI (0.210 ± 0.041 vs. −0.190 ± 0.028; *p* < 0.001; Figure 2F). After adjustment for age, sex, systolic blood pressure, BMI, diabetes mellitus, and a history of cerebrovascular disease, a higher PBUT score remained independently associated with MCI (coefficient, 0.290 ± 0.077; odds ratio, 1.337; 95% confidence interval, 1.151–1.554; *p* < 0.001; Hosmer–Lemeshow χ^2^ = 8.668; Table 2). Because of the cross-sectional design of the present study, the observed associations cannot establish causality or distinguish causation from reverse causation.

In prespecified secondary analyses of individual biomarkers and kidney function measures, similar associations for IS (coefficient, 0.265 ± 0.078; odds ratio, 1.304; 95% confidence interval, 1.118–1.520; *p* < 0.001; Hosmer–Lemeshow χ^2^ = 9.008; Table S2), PCS (coefficient, 0.232 ± 0.075; odds ratio, 1.261; 95% confidence interval, 1.089–1.461; *p* = 0.002; Hosmer–Lemeshow χ^2^ = 11.026; Table S3), IAA (coefficient, 0.293 ± 0.082; odds ratio, 1.341; 95% confidence interval, 1.141–1.575; *p* < 0.001; Hosmer–Lemeshow χ^2^ = 12.872; Table S4), PhS (coefficient, 0.109 ± 0.075; odds ratio, 1.115; 95% confidence interval, 0.962–1.292; *p* = 0.147; Hosmer–Lemeshow χ^2^ = 8.825; Table S5), HA (coefficient, 0.206 ± 0.072; odds ratio, 1.228; 95% confidence interval, 1.065–1.416; Hosmer–Lemeshow χ^2^ = 8.668; Table S6), β2-m (coefficient, 0.293 ± 0.074; odds ratio, 1.341; 95% confidence interval, 1.160–1.550; Hosmer–Lemeshow χ^2^ = 9.792; Table S7), GFRcre (coefficient, 0.184 ± 0.071; odds ratio, 1.202; 95% confidence interval, 1.046–1.382; *p* = 0.009; Hosmer–Lemeshow χ^2^ = 7.945; Table S8), and GFRcys (coefficient, 0.269 ± 0.051; odds ratio, 1.309; 95% confidence interval, 1.137–1.507; *p* < 0.001; Hosmer–Lemeshow χ^2^ = 11.339; Table S9). DeLong’s test revealed no significant pairwise differences in AUC among the models incorporating the PBUT score, GFRcys, or GFRcre (all *p* ≥ 0.05). The corresponding AUCs were 0.800, 0.799, and 0.797, and the Youden indices were 0.468, 0.461, and 0.459, respectively, indicating comparable discriminative performance (Table S10).

### 2.5. Sensitivity Analysis

Sensitivity analyses yielded results consistent with the primary analysis. First, after additional adjustment for GFRcre, the association between the PBUT score and cognitive impairment remained similar (Table S11). Second, to assess internal reproducibility, the dataset was randomly divided into a training set (*n* = 608) and a validation set (*n* = 609); the association between the PBUT score and MMSE-defined cognitive impairment was similar in both datasets (Table S12). Third, stratified analyses showed similar associations between the PBUT score and cognitive impairment among participants with early-stage and advanced-stage CKD (Table S13).

Because the primary analysis defined possible MCI using an MMSE cutoff of <28, we assessed the robustness of the findings using alternative outcome definitions. When severe cognitive impairment was defined as an MMSE score of <23, the PBUT score remained independently associated with cognitive impairment (Table S14). In addition, when the MMSE score was analyzed as a continuous variable, a higher PBUT score was associated with a lower MMSE score (Table S15).

## 3. Discussion

This cross-sectional analysis of a community-based cohort comprehensively evaluated PBUTs and cognitive function assessed using the MMSE and identified an association between the PBUT score and MCI. This association remained consistent across alternative definitions of cognitive impairment; however, the cross-sectional design precludes establishing temporality or causality and does not exclude reverse causation.

Consistent with findings from previous systematic reviews and meta-analyses, the results showed that more advanced CKD stages were associated with lower MMSE scores. Zhang et al. summarized data from 50 studies involving 25,289 patients with CKD and reported prevalences of cognitive impairment of 32%, 53%, and 39% among patients with predialysis CKD, hemodialysis (HD), and peritoneal dialysis, respectively [7]. These findings suggest that cognitive impairment is present across the spectrum of CKD and is more frequent in some advanced-stage populations. Therefore, early identification and management of CKD-associated cognitive impairment are essential.

Multiple factors contribute to cognitive impairment in patients with CKD. In addition to CKD progression, traditional risk factors such as advanced age and a history of diabetes mellitus or hypertension are involved [5]. Other CKD-related factors include gut-derived uremic toxins; metabolic disturbances, such as metabolic acidosis and hyponatremia; hormonal imbalances, such as elevated parathyroid hormone levels, vitamin D deficiency, and anemia; systemic inflammation characterized by oxidative stress and elevated inflammatory cytokines; and polypharmacy or specific medications [5].

Among these, PBUTs represent potential clinical factors that may directly impair cognitive function [6]; however, evidence regarding the specific relationship between PBUTs and cognitive impairment remains limited. A cross-sectional study of patients undergoing HD demonstrated an association between serum PCS levels and cognitive function [18]. Metabolomic analyses of plasma from patients receiving HD identified associations between impaired executive function and 4-hydroxyphenylacetate, phenylacetylglutamine, hippurate, and prolyl-hydroxyproline [19]. Among maintenance HD patients with depression, serum IS levels, but not PCS levels, were lower than those observed in patients without depressive symptoms [20]. Nevertheless, few studies have investigated the relationship between PBUTs and cognitive function in populations that include patients with nondialysis CKD. Yeh et al. conducted a cross-sectional study of 199 patients with CKD stages 3 to 5 and found that executive function in stage 3 CKD was associated with IS levels, but not PCS levels [21].

In the present study, the composite PBUT score was associated with cognitive function across CKD stages after adjustment for age, sex, blood pressure, diabetes, and a history of cerebrovascular disease. Although the primary analysis used an MMSE cutoff of <28 to identify possible MCI, the findings remained consistent when severe cognitive impairment was defined using a cutoff of <23 and when MMSE scores were analyzed as a continuous variable. This consistency across alternative outcome definitions supports the robustness of the association between a higher PBUT score and poorer cognitive function.

Serum PBUT concentrations increase as kidney function declines, and experimental evidence indicates that these molecules can accumulate in tissues and contribute to organ dysfunction. In an adenine-induced kidney injury mouse model, Sato et al. detected accumulation of PBUTs, including IS, PCS, and PhS, in brain tissue using mass spectrometry, and treatment with the oral charcoal adsorbent AST-120 reduced this cerebral accumulation [13]. These experimental findings suggest that PBUTs accumulating during kidney dysfunction may reach brain tissue in experimental models; however, the present cross-sectional study cannot establish this mechanism in humans.

In vitro evidence indicates that serum from dialysis patients promotes inflammatory responses in astrocytes in an IS concentration-dependent manner [22]. Similarly, Watanabe et al. reported decreased viability of neuronal HT-22 cells following exposure to IS, PCS, or PhS [23]. Additionally, IS and PCS are known to promote vascular calcification and atherosclerosis [24,25]. Collectively, these findings suggest that various PBUTs elevated in kidney dysfunction may accumulate in the brain parenchyma, exert direct neurotoxic effects, or be associated with impaired microvascular integrity, and thereby potentially be related to cognitive decline. While our findings provide clinical support for a potential link between PBUT burden and cognitive impairment, they do not establish causality or definitively prove these mechanisms. Whether interventions targeting PBUT accumulation can help prevent cognitive decline requires prospective clinical evaluation.

In the present study, the composite PBUT score was associated with cognitive function across CKD stages. By capturing the combined burden of several correlated toxins, a comprehensive assessment of PBUTs may complement conventional measures of kidney function. A previous study demonstrated an association between the PBUT score and pruritus in dialysis patients [26], and the present findings extend this observational association to cognitive impairment. In the multivariable logistic regression analysis, the association between PhS and cognitive function was weaker than those observed for the other PBUTs. Cognitive impairment may reflect the cumulative burden of multiple PBUTs rather than the independent effect of any single toxin. Thus, although some individual PBUTs, including PhS, were not significantly associated with cognitive impairment, the composite PBUT score may better reflect overall exposure to multiple toxins. Models incorporating the PBUT score, GFRcys stage, or GFRcre stage showed comparable discriminative performance, with no significant differences in AUC. Nevertheless, a higher PBUT burden was associated with cognitive impairment independently of several established clinical factors. Prospective studies are needed to determine whether the PBUT score provides incremental predictive value beyond measures of kidney function and whether PBUTs play a causal role in cognitive decline.

This study has several limitations that warrant consideration. First, due to its cross-sectional design, temporal or causal relationships between PBUT levels and cognitive impairment cannot be established, and reverse causation remains a possibility. Furthermore, residual confounding from unmeasured variables cannot be ruled out; in particular, detailed information associated with cognitive functions, such as medication records, educational level, depression, anemia, socioeconomic status, alcohol use, and other neurological or psychiatric conditions were unavailable in this cohort. Second, although our findings suggest the potential utility of the PBUT score as a composite indicator of overall PBUT burden, formal validation was not performed in this analysis. Consequently, its clinical applicability, reproducibility, external validity, and incremental value beyond established kidney function measures require confirmation in independent prospective cohorts. Third, our primary outcome focused on possible MCI to emphasize early detection and prevention. Although sensitivity analyses encompassed more severe cognitive impairment, this focus may limit the generalizability of our findings across the full spectrum of cognitive disorders. Despite these limitations, this study identifies an association between composite PBUT burden and cognitive impairment in a large, community-based population.

## 4. Conclusions

Lower cognitive performance was associated with higher PBUT levels and more advanced kidney dysfunction in this community-based cohort. These findings identify PBUT burden as a potential correlate of cognitive impairment in CKD. Prospective studies are needed to clarify its temporal relationship with cognitive decline, assess its predictive value beyond established measures of kidney function, and determine whether interventions targeting PBUTs affect cognitive outcomes.

## 5. Materials and Methods

### 5.1. Study Design

This cross-sectional study analyzed participants from the PROject in Sado for Total Health (PROST), a community-based cohort conducted in Sado City, Niigata Prefecture, Japan [14]. PROST enrolled 3295 individuals between May 2008 and December 2017. Of these, 1217 who underwent MMSE assessment and had measurements of PBUTs, GFRcys, GFRcre, and β2-m were included in the present analysis (Figure S3). The study conformed to the principles of the Declaration of Helsinki and was approved by the Central Ethics Committee of Niigata University (No. 2018-0246). The study was registered with the University Hospital Medical Information Network Clinical Trials Registry (UMIN000034240). All participants provided written informed consent for the original PROST study, and an opt-out procedure was used for the present analysis.

### 5.2. Patient Characteristics

Demographic and clinical data, including age, sex, BMI, systolic and diastolic blood pressure, diabetes mellitus, histories of cardiovascular and cerebrovascular events, and MMSE scores, were obtained from the PROST database [14].

Serum specimens collected from patients were immediately frozen at −30 °C and thawed immediately before PBUT measurement. Levels of the total forms of five PBUTs, IS, PCS, IAA, PhS, and HA, were measured using mass spectrometry, as previously described [8]. Serum creatinine levels were measured using the improved Jaffe method (DICT-500; BioAssay Systems, Hayward, CA, USA), and GFR based on standardized serum creatinine (GFRcre) was calculated according to an established formula [15]. Serum cystatin C levels were measured using enzyme-linked immunosorbent assay (DSCTC0; R&D Systems, Inc., Minneapolis, MN, USA) and estimated GFR based on standardized serum cystatin C (GFRcys) was calculated as described in previous reports [16]. Serum β2-m levels were also measured using enzyme-linked immunosorbent assay (KGE019; R&D Systems, Inc.).

### 5.3. Statistical Analysis

Results are expressed as mean ± standard deviation or frequency (%) for descriptive purposes. Participants with MMSE scores < 28 were classified as having possible mild cognitive impairment for the primary analysis, based on previous reports [16,17]. Differences between groups with and without possible mild cognitive impairment were assessed using an unpaired *t*-test or Mann–Whitney U-test for continuous variables and a χ^2^ test for categorical variables. A composite PBUT score was derived from standardized concentrations of IS, PCS, PhS, IAA, and HA using principal component analysis [21]. Continuous variables used in the regression analyses, including individual PBUTs, β2-m, GFRcre, GFRcys, age, systolic blood pressure, and BMI, were standardized before analysis. CKD stages were categorized according to GFR as G1 (≥90 mL/min/1.73 m^2^), G2 (60–89 mL/min/1.73 m^2^), G3a (45–59 mL/min/1.73 m^2^), G3b (30–44 mL/min/1.73 m^2^), G4 (15–29 mL/min/1.73 m^2^), and G5 (<15 mL/min/1.73 m^2^). Principal component analysis reduces multiple correlated variables into fewer factors. Two-sample statistical tests for each PBUT and the principal component score derived from all PBUTs were conducted to examine independent predictors of cognitive impairment. Univariate and multivariate logistic regression analyses were performed, with the presence or absence of cognitive impairment as the dependent variable. In the primary analysis, the association between the composite PBUT score and possible MCI, defined as an MMSE score of <28, was examined. Prespecified secondary analyses examined the associations of possible MCI with each individual PBUT, β2-m, GFRcys, and GFRcre. Independent variables included PBUT score, age, sex, systolic blood pressure, BMI, diabetes mellitus, and history of cerebrovascular disease. Model fit for the multivariate logistic regression models was evaluated using Hosmer-Lemeshow test. The χ^2^ value by Hosmer-Lemeshow test is a measure with smaller values indicating a better fit to the data. Discrimination of models including GFRcre stage, GFRcys stage, or the PBUT score was assessed using ROC curves based on predicted probabilities from multivariable logistic regression models adjusted for the same background characteristics, including age and sex. AUCs were compared pairwise using DeLong’s method. For each model, the optimal cutoff maximized the Youden index (sensitivity + specificity − 1). As a supplementary reference measure, a composite index was calculated by summing the standardized regression coefficient and the reciprocal of the Hosmer-Lemeshow good-ness-of-fit chi-square statistic. This exploratory index was intended to provide an integrated summary of both the magnitude of the predictor effect and the overall model fit. A higher value for this composite indicator suggests a greater effect of the prognostic factors and a better overall model fit. The composite index was used solely as a supplementary reference for model comparison and was not intended to replace established measures of model performance. A two-sided *p* < 0.05 was considered statistically significant. Sensitivity analyses were conducted as follows. First, the primary multivariable logistic regression analysis was repeated with additional adjustment for GFRcre. Second, to assess the internal reproducibility of the findings, the dataset was randomly divided into a training set (*n* = 608) and a validation set (*n* = 609), and the association between the PBUT score and possible MCI was examined separately in each set. Third, analyses were stratified by CKD severity to examine this association among participants with early-stage CKD (GFRcre ≥ 60 mL/min/1.73 m^2^) and advanced-stage CKD (GFRcre < 60 mL/min/1.73 m^2^). Fourth, the logistic regression analyses were repeated using severe cognitive impairment, defined as an MMSE score of <23 based on previous reports [27,28], as an alternative outcome. Finally, linear regression analysis was used to evaluate the association between the PBUT score and MMSE score as a continuous outcome.

## Figures and Tables

**Table 1:** Characteristics of participants.

	All *n* = 1217	MMSE < 28 *n* = 587	MMSE ≥ 28 *n* = 630	*p* Value
MMSE	26.7 ± 3.4	23.9 ± 2.8	29.2 ± 0.8	<0.001 †
Age, years	65.5 ± 12.1	71.4 ± 9.5	60.0 ± 11.7	<0.001 †
Male sex, %	52.7	57.1	48.6	0.003 ‡
Body mass index, kg/m^2^	23.9 ± 4.0	23.6 ± 3.9	24.2 ± 4.1	0.015 †
Systolic blood pressure, mmHg	137.5 ± 29.9	138.4 ± 19.9	136.6 ± 36.8	0.272 †
Diastolic blood pressure, mmHg	75.6 ± 13.1	74.1 ± 12.8	76.92 ± 13.3	<0.001 †
Diabetes, %	34.1	31.5	36.5	0.070 ‡
Myocardial infarction, %	5.0	6.5	3.7	0.026 ‡
Cerebrovascular disease, %	12.1	18.1	6.5	<0.001 ‡
GFRcre, mL/min/1.73 m^2^	79.6 ± 79.4	72.8 ± 76.4	85.8 ± 81.7	0.004 †
GFRcys, mL/min/1.73 m^2^	60.5 ± 26.5	53.1 ± 25.4	67.4 ± 25.6	<0.001 †
b_2_-microglobulin, mg/L	4.0 ± 6.1	4.9 ± 6.9	3.3 ± 5.2	<0.001 †

GFRcre, glomerular filtration rate for creatinine; GFRcys, glomerular filtration rate for cystatin C; MMSE, Mini-Mental State Examination. †: *p* values were calculated by *t*-tests. ‡: *p* values were calculated by χ^2^ tests.

**Table 2:** Association of cognitive impairment with protein-bound uremic toxin score and clinical factors.

	Univariate						Multivariate						Hosmer-Lemeshow Test	
Independent Variables	Coefficient †	SE	OR	OR [Lower]	OR [Upper]	*p* Value	Coefficient †	SE	OR	OR [Lower]	OR [Upper]	*p* Value	χ^2^ ‡	*p* Value §
Protein-bound uremic toxin score	0.303	0.066	1.353	1.189	1.540	<0.001	0.290	0.077	1.337	1.151	1.554	<0.001	8.668	0.371
Male gender	0.342	0.115	1.408	1.123	1.765	0.003	0.396	0.138	1.485	1.132	1.948	0.004		
Age	1.278	0.087	3.588	3.024	4.258	<0.001	1.327	0.094	3.769	3.135	4.530	<0.001		
Systolic blood pressure	0.069	0.066	1.072	0.942	1.220	0.294	−0.058	0.079	0.943	0.808	1.101	0.461		
Body mass index	−0.143	0.059	0.867	0.773	0.973	0.015	0.183	0.075	1.201	1.038	1.390	0.014		
Diabetes mellitus	−0.223	0.121	0.800	0.631	1.015	0.067	0.075	0.145	1.078	0.812	1.431	0.603		
History of cerebrovascular disease	1.152	0.194	3.166	2.165	4.630	<0.001	0.754	0.219	2.125	1.384	3.263	<0.001		

† Standardized coefficient; larger absolute values indicate stronger associations. ‡ χ^2^ statistic from the Hosmer–Lemeshow goodness-of-fit test. § *p* value from the Hosmer–Lemeshow goodness-of-fit test; a non-significant value indicates no evidence of poor model fit.

## Data Availability

The data presented in this study are available on request from the corresponding author due to privacy.

## Supplementary Materials

The following supporting information can be downloaded at: https://www.mdpi.com/article/10.3390/toxins18100422/s1, Table S1: Principal component analysis for protein-bound uremic toxins; Table S2: Association of mild cognitive impairment with serum indoxyl sulfate level and clinical factors; Table S3: Association of mild cognitive impairment with serum p-cresyl sulfate level and clinical factors; Table S4: Association of mild cognitive impairment with serum indole acetic acid level and clinical factors; Table S5: Association of mild cognitive impairment with serum phenyl sulfate level and clinical factors; Table S6: Association of mild cognitive impairment with serum hippuric acid level and clinical factors; Table S7: Association of mild cognitive impairment with serum β_2_-microglobulin level and clinical factors; Table S8: Association of mild cognitive impairment with GFRcre and clinical factors; Table S9: Association of mild cognitive impairment with GFRcys and clinical factors; Table S10: Effect of protein-bound uremic toxin score, GFRcre and GFRcys on cognitive function; Table S11: Association of mild cognitive impairment with protein-bound uremic toxin score and clinical factors including GFRcre; Table S12: Internal validation analysis of association of MMSE with protein-bound uremic toxin score and clinical factors; Table S13: Association of cognitive impairment, MMSE <23, with protein-bound uremic toxin score and clinical factors stratified with CKD severity; Table S14: Association of cognitive impairment, MMSE <23, with protein-bound uremic toxin score and clinical factors; Table S15: Association of continuous MMSE scores with protein-bound uremic toxin score and clinical factors; Figure S1: Protein-bound uremic toxins according to kidney dysfunction severity; Figure S2: Scree plot for principal component analysis of protein-bound uremic toxins; Figure S3: Flow diagram of participant selection.

## Abbreviations

The following abbreviations are used in this manuscript:

ADL	activities of daily living
BMI	body mass index
CKD	chronic kidney disease
GFR	glomerular filtration rate
GFRcre	glomerular filtration rate based on standardized serum creatinine
GFRcys	glomerular filtration rate based on standardized serum cystatin C
ESKD	end-stage kidney disease
HA	hippuric acid
HD	hemodialysis
IAA	indole acetic acid
IS	indoxyl sulfate
MCI	mild cognitive impairment
MMSE	mini-mental state examination
PBUTs	protein-bound uremic toxins
PCS	p-cresyl sulfate
PhS	phenyl sulfate
PROST	PROject in Sado for Total Health
QOL	quality of life
β2-m	β_2_-microglobulin
