# Supplementary material for: Protein-Bound Uremic Toxins and Cognitive Function: A Cross-Sectional Study in a Community-Based Cohort
Source: Toxins (Basel). 2026 Sep 30;18(10):422. doi: 10.3390/toxins18100422 (PMC13709565; doi:10.3390/toxins18100422)

Supplemental Materials

Protein-Bound Uremic Toxins and Cognitive Function: A Cross-sectional Study in a Community-based Cohort

Suguru Yamamoto, Takahiro Tanaka, Nobutaka Kitamura, Miho Kawachi, Akio Yokoseki, Minako Wakasugi, Osamu Onodera, Naoto Endo, Masakazu Wada, Kenji Sato, Kaori Kikuchi, Ayano Konagai, Shin Goto, Ichiei Narita

Table S1. Principal component analysis for protein-bound uremic toxins

Principal component	Eigen value	Proportion	Cumulative proportion
1	3.035	60.7%	60.7%
2	0.739	14.8%	75.5%
3	0.645	12.9%	88.4%

Factor extraction method: Maximum likelihood method

Table S2. Association of mild cognitive impairment with serum indoxyl sulfate level and clinical factors

	Univariate						Multivariate						Hosmer-Lemeshow test	
Independent variables	Coefficient†	SE	OR	OR [Lower]	OR [Upper]	P value	Coefficient†	SE	OR	OR [Lower]	OR [Upper]	P value	χ^2‡	P value §
Indoxyl sulfate	0.221	0.064	1.247	1.099	1.415	<.001	0.265	0.078	1.304	1.118	1.520	<.001	9.008	0.342
Male gender	0.342	0.115	1.408	1.123	1.765	0.003	0.411	0.138	1.508	1.150	1.976	0.003		
Age	1.278	0.087	3.588	3.024	4.258	<.001	1.338	0.094	3.811	3.168	4.583	<.001		
Systolic blood pressure	0.069	0.066	1.072	0.942	1.220	0.294	-0.052	0.077	0.949	0.816	1.105	0.501		
Body mass index	-0.143	0.059	0.867	0.773	0.973	0.015	0.166	0.074	1.181	1.021	1.365	0.025		
Diabetes mellitus	-0.223	0.121	0.800	0.631	1.015	0.067	0.100	0.144	1.105	0.833	1.467	0.487		
History of cerebrovascular disease	1.152	0.194	3.166	2.165	4.630	<.001	0.770	0.218	2.161	1.408	3.315	<.001		

†: Standardized coefficient; the higher the value, the greater the influence.

‡: Chi-square value from the Hosmer-Lemeshow test; the lower the value, the better the fit.

§: P value from the Hosmer-Lemeshow test; the higher the value, the better the fit.

Table S3. Association of mild cognitive impairment with serum p-cresyl sulfate level and clinical factors

	Univariate						Multivariate						Hosmer-Lemeshow test	
Independent variables	Coefficient†	SE	OR	OR [Lower]	OR [Upper]	P value	Coefficient†	SE	OR	OR [Lower]	OR [Upper]	P value	χ^2‡	P value §
p-Cresyl sulfate	0.342	0.068	1.408	1.233	1.608	<.001	0.232	0.075	1.261	1.089	1.461	0.002	11.026	0.200
Male gender	0.342	0.115	1.408	1.123	1.765	0.003	0.420	0.138	1.522	1.161	1.995	0.002		
Age	1.278	0.087	3.588	3.024	4.258	<.001	1.297	0.093	3.657	3.047	4.390	<.001		
Systolic blood pressure	0.069	0.066	1.072	0.942	1.220	0.294	-0.035	0.071	0.965	0.839	1.110	0.622		
Body mass index	-0.143	0.059	0.867	0.773	0.973	0.015	0.160	0.074	1.173	1.015	1.357	0.031		
Diabetes mellitus	-0.223	0.121	0.800	0.631	1.015	0.067	0.036	0.145	1.036	0.780	1.378	0.806		
History of cerebrovascular disease	1.152	0.194	3.166	2.165	4.630	<.001	0.747	0.218	2.110	1.376	3.236	<.001		

†: Standardized coefficient; the higher the value, the greater the influence.

‡: Chi-square value from the Hosmer-Lemeshow test; the lower the value, the better the fit.

§: P value from the Hosmer-Lemeshow test; the higher the value, the better the fit.

Table S4. Association of mild cognitive impairment with serum indole acetic acid level and clinical factors

	Univariate						Multivariate						Hosmer-Lemeshow test	
Independent variables	Coefficient†	SE	OR	OR [Lower]	OR [Upper]	P value	Coefficient†	SE	OR	OR [Lower]	OR [Upper]	P value	χ^2‡	P value §
Indole acetic acid	0.429	0.078	1.535	1.319	1.787	<.001	0.293	0.082	1.341	1.141	1.575	<.001	12.872	0.116
Male gender	0.342	0.115	1.408	1.123	1.765	0.003	0.39	0.139	1.476	1.125	1.937	0.005		
Age	1.278	0.087	3.588	3.024	4.258	<.001	1.299	0.094	3.664	3.049	4.403	<.001		
Systolic blood pressure	0.069	0.066	1.072	0.942	1.220	0.294	-0.036	0.071	0.964	0.839	1.108	0.61		
Body mass index	-0.143	0.059	0.867	0.773	0.973	0.015	0.169	0.074	1.184	1.024	1.368	0.022		
Diabetes mellitus	-0.223	0.121	0.800	0.631	1.015	0.067	0.061	0.145	1.062	0.800	1.411	0.676		
History of cerebrovascular disease	1.152	0.194	3.166	2.165	4.630	<.001	0.725	0.22	2.064	1.342	3.175	<.001		

†: Standardized coefficient; the higher the value, the greater the influence.

‡: Chi-square value from the Hosmer-Lemeshow test; the lower the value, the better the fit.

§: P value from the Hosmer-Lemeshow test; the higher the value, the better the fit.

Table S5. Association of mild cognitive impairment with serum phenyl sulfate level and clinical factors

	Univariate						Multivariate						Hosmer-Lemeshow test	
Independent variables	Coefficient†	SE	OR	OR [Lower]	OR [Upper]	P value	Coefficient†	SE	OR	OR [Lower]	OR [Upper]	P value	χ^2‡	P value §
Phenyl sulfate	0.170	0.068	1.185	1.038	1.353	0.012	0.109	0.075	1.115	0.962	1.292	0.147	8.825	0.357
Male gender	0.342	0.115	1.408	1.123	1.765	0.003	0.424	0.138	1.528	1.167	2.001	0.002		
Age	1.278	0.087	3.588	3.024	4.258	<.001	1.312	0.093	3.712	3.092	4.457	<.001		
Systolic blood pressure	0.069	0.066	1.072	0.942	1.220	0.294	-0.014	0.066	0.986	0.866	1.123	0.832		
Body mass index	-0.143	0.059	0.867	0.773	0.973	0.015	0.123	0.073	1.131	0.980	1.304	0.091		
Diabetes mellitus	-0.223	0.121	0.800	0.631	1.015	0.067	0.093	0.144	1.097	0.828	1.454	0.517		
History of cerebrovascular disease	1.152	0.194	3.166	2.165	4.630	<.001	0.772	0.218	2.165	1.413	3.317	<.001		

†: Standardized coefficient; the higher the value, the greater the influence.

‡: Chi-square value from the Hosmer-Lemeshow test; the lower the value, the better the fit.

§: P value from the Hosmer-Lemeshow test; the higher the value, the better the fit.

Table S6. Association of mild cognitive impairment with serum hippuric acid level and clinical factors

	Univariate						Multivariate						Hosmer-Lemeshow test	
Independent variables	Coefficient	SE	OR	OR [Lower]	OR [Upper]	P value	Coefficient	SE	OR	OR [Lower]	OR [Upper]	P value	χ^2‡	P value §
Hippuric acid	0.108	0.061	1.114	0.989	1.255	0.075	0.206	0.072	1.228	1.065	1.416	0.005	10.142	0.255
Male gender	0.342	0.115	1.408	1.123	1.765	0.003	0.423	0.138	1.527	1.166	2.000	0.002		
Age	1.278	0.087	3.588	3.024	4.258	<.001	1.345	0.095	3.837	3.187	4.618	<.001		
Systolic blood pressure	0.069	0.066	1.072	0.942	1.220	0.294	-0.041	0.074	0.960	0.830	1.109	0.575		
Body mass index	-0.143	0.059	0.867	0.773	0.973	0.015	0.158	0.074	1.172	1.013	1.355	0.032		
Diabetes mellitus	-0.223	0.121	0.800	0.631	1.015	0.067	0.106	0.144	1.112	0.838	1.475	0.461		
History of cerebrovascular disease	1.152	0.194	3.166	2.165	4.630	<.001	0.781	0.218	2.183	1.423	3.349	<.001		

†: Standardized coefficient; the higher the value, the greater the influence.

‡: Chi-square value from the Hosmer-Lemeshow test; the lower the value, the better the fit.

§: P value from the Hosmer-Lemeshow test; the higher the value, the better the fit.

Table S7. Association of mild cognitive impairment with serum β_2-microglobulin level and clinical factors

	Univariate						Multivariate						Hosmer-Lemeshow test	
Independent variables	Coefficient†	SE	OR	OR [Lower]	OR [Upper]	P value	Coefficient†	SE	OR	OR [Lower]	OR [Upper]	P value	χ^2‡	P value §
β_2-microglobulin	0.275	0.063	1.317	1.164	1.490	<.001	0.293	0.074	1.341	1.160	1.550	<.001	9.792	0.280
Male gender	0.342	0.115	1.408	1.123	1.765	0.003	0.413	0.138	1.511	1.153	1.981	0.003		
Age	1.278	0.087	3.588	3.024	4.258	<.001	1.335	0.094	3.798	3.157	4.569	<.001		
Systolic blood pressure	0.069	0.066	1.072	0.942	1.220	0.294	-0.064	0.081	0.938	0.800	1.099	0.428		
Body mass index	-0.143	0.059	0.867	0.773	0.973	0.015	0.184	0.075	1.202	1.038	1.391	0.014		
Diabetes mellitus	-0.223	0.121	0.800	0.631	1.015	0.067	0.081	0.145	1.085	0.817	1.441	0.573		
History of cerebrovascular disease	1.152	0.194	3.166	2.165	4.630	<.001	0.745	0.219	2.107	1.371	3.239	<.001		

†: Standardized coefficient; the higher the value, the greater the influence.

‡: Chi-square value from the Hosmer-Lemeshow test; the lower the value, the better the fit.

§: P value from the Hosmer-Lemeshow test; the higher the value, the better the fit.

Table S8. Association of mild cognitive impairment with GFRcre and clinical factors

	Univariate						Multivariate						Hosmer-Lemeshow test	
Independent variables	Coefficient†	SE	OR	OR [Lower]	OR [Upper]	P value	Coefficient†	SE	OR	OR [Lower]	OR [Upper]	P value	χ^2‡	P value §
GFRcre	0.328	0.060	1.388	1.235	1.560	<.001	0.184	0.071	1.202	1.046	1.382	0.009	7.945	0.439
Male gender	0.342	0.115	1.408	1.123	1.765	0.003	0.412	0.138	1.509	1.152	1.978	0.003		
Age	1.278	0.087	3.588	3.024	4.258	<.001	1.294	0.093	3.649	3.038	4.383	<.001		
Systolic blood pressure	0.069	0.066	1.072	0.942	1.220	0.294	-0.033	0.072	0.968	0.840	1.116	0.653		
Body mass index	-0.143	0.059	0.867	0.773	0.973	0.015	0.156	0.074	1.169	1.010	1.353	0.036		
Diabetes mellitus	-0.223	0.121	0.800	0.631	1.015	0.067	0.072	0.144	1.075	0.810	1.425	0.618		
History of cerebrovascular disease	1.152	0.194	3.166	2.165	4.630	<.001	0.755	0.218	2.128	1.389	3.260	<.001		

GFRcre, glomerular filtration rate estimated using standardized serum creatinine. CKD G categories based on GFRcre were defined as G1 (≥90), G2 (60–89), G3a (45–59), G3b (30–44), G4 (15–29), and G5 (<15 mL/min/1.73 m^2).

†: Standardized coefficient; the higher the value, the greater the influence.

‡: Chi-square value from the Hosmer-Lemeshow test; the lower the value, the better the fit.

§: P value from the Hosmer-Lemeshow test; the higher the value, the better the fit.

Table S9. Association of mild cognitive impairment with GFRcys and clinical factors

	Univariate						Multivariate						Hosmer-Lemeshow test	
Independent variables	Coefficient†	SE	OR	OR [Lower]	OR [Upper]	P value	Coefficient†	SE	OR	OR [Lower]	OR [Upper]	P value	χ^2‡	P value §
GFRcys	0.518	0.063	1.678	1.482	1.9	<.001	0.269	0.072	1.309	1.137	1.507	<.001	11.339	0.183
Male gender	0.342	0.115	1.408	1.123	1.765	0.003	0.413	0.138	1.512	1.153	1.982	0.003		
Age	1.278	0.087	3.588	3.024	4.258	<.001	1.253	0.094	3.501	2.911	4.210	<.001		
Systolic blood pressure	0.069	0.066	1.072	0.942	1.22	0.294	-0.041	0.074	0.959	0.830	1.109	0.576		
Body mass index	-0.143	0.059	0.867	0.773	0.973	0.015	0.158	0.073	1.171	1.014	1.352	0.031		
Diabetes mellitus	-0.223	0.121	0.800	0.631	1.015	0.067	0.077	0.144	1.080	0.813	1.433	0.595		
History of cerebrovascular disease	1.152	0.194	3.166	2.165	4.63	<.001	0.759	0.218	2.137	1.393	3.277	<.001		

GFRcys, glomerular filtration rate estimated using standardized serum cystatin C. CKD G categories based on GFRcys were defined as G1 (≥90), G2 (60–89), G3a (45–59), G3b (30–44), G4 (15–29), and G5 (<15 mL/min/1.73 m^2).

†: Standardized coefficient; the higher the value, the greater the influence.

‡: Chi-square value from the Hosmer-Lemeshow test; the lower the value, the better the fit.

§: P value from the Hosmer-Lemeshow test; the higher the value, the better the fit.

Table S10. Effect of protein-bound uremic toxin score, GFRcre and GFRcys on cognitive function

Independent variables	AUC	SE	Lower	Upper	Asymptotic P value	Youden Index
Protein-bound uremic toxin score	0.800	0.013	0.775	0.824	0.000	0.468
GFRcre	0.797	0.013	0.772	0.822	0.000	0.459
GFRcys	0.799	0.013	0.774	0.823	0.000	0.461

GFRcre; glomerular filtration rate estimated using standardized serum creatinine

GFRcys; glomerular filtration rate estimated using standardized serum cystatin C

Table S11. Association of mild cognitive impairment with protein-bound uremic toxin score and clinical factors including GFRcre

	Multivariate						Hosmer-Lemeshow test	
Independent variables	Coefficient†	SE	OR	OR [Lower]	OR [Upper]	P value	χ^2‡	P value §
Protein-bound uremic toxin score	0.296	0.104	1.344	1.097	1.648	0.004	9.175	0.328
Male gender	0.396	0.139	1.486	1.132	1.949	0.004		
Age	1.328	0.095	3.772	3.133	4.542	<.001		
Systolic blood pressure	-0.058	0.079	0.944	0.808	1.102	0.463		
Body mass index	0.183	0.075	1.200	1.036	1.390	0.015		
Diabetes mellitus	0.076	0.145	1.079	0.812	1.432	0.601		
History of cerebrovascular disease	0.754	0.219	2.126	1.384	3.264	<.001		
GFRcre	-0.008	0.097	0.992	0.820	1.201	0.936		

GFRcre; glomerular filtration rate estimated using standardized serum creatinine

‡: Chi-square value from the Hosmer-Lemeshow test; the lower the value, the better the fit.

§: P value from the Hosmer-Lemeshow test; the higher the value, the better the fit.

Table S12. Internal validation analysis of association of MMSE with protein-bound uremic toxin score and clinical factors

Training dataset (n=608)

	Multivariate						Hosmer-Lemeshow test	
Independent variables	Coefficient†	SE	OR	OR [Lower]	OR [Upper]	P value	χ^2‡	P value §
Protein-bound uremic toxin score	0.290	0.11	1.337	1.077	1.659	0.008	11.365	0.182
Male gender	0.354	0.198	1.425	0.966	2.103	0.074		
Age	1.346	0.135	3.844	2.947	5.012	<.001		
Systolic blood pressure	-0.208	0.143	0.812	0.614	1.074	0.145		
Body mass index	0.308	0.104	1.361	1.11	1.668	0.003		
Diabetes mellitus	0.129	0.206	1.138	0.76	1.702	0.53		
History of cerebrovascular disease	0.956	0.337	2.602	1.344	5.037	0.005		

Validation dataset (n=609)

	Multivariate						Hosmer-Lemeshow test	
Independent variables	Coefficient†	SE	OR	OR [Lower]	OR [Upper]	P value	χ^2‡	P value §
Protein-bound uremic toxin score	0.251	0.111	1.285	1.035	1.597	0.023	7.522	0.481
Male gender	0.394	0.197	1.483	1.008	2.179	0.045		
Age	1.314	0.133	3.722	2.87	4.828	<.001		
Systolic blood pressure	0.164	0.151	1.178	0.877	1.584	0.277		
Body mass index	0.046	0.108	1.047	0.848	1.294	0.668		
Diabetes mellitus	0.019	0.207	1.019	0.679	1.528	0.928		
History of cerebrovascular disease	0.584	0.292	1.793	1.012	3.176	0.045		

‡: Chi-square value from the Hosmer-Lemeshow test; the lower the value, the better the fit.

§: P value from the Hosmer-Lemeshow test; the higher the value, the better the fit.

Table S13. Association of cognitive impairment, MMSE <23, with protein-bound uremic toxin score and clinical factors stratified with CKD severity

GFRcre ≥ 60 mL/min/1.73m^2

	Multivariate						Hosmer-Lemeshow test	
Independent variables	Coefficient†	SE	OR	OR [Lower]	OR [Upper]	P value	χ^2‡	P value §
Protein-bound uremic toxin score	1.141	0.453	3.129	1.287	7.611	0.012	7.029	0.534
Male gender	0.569	0.178	1.766	1.246	2.503	0.001		
Age	1.279	0.124	3.594	2.820	4.581	<.001		
Systolic blood pressure	-0.043	0.084	0.958	0.813	1.130	0.612		
Body mass index	0.136	0.092	1.146	0.957	1.373	0.139		
Diabetes mellitus	0.032	0.188	1.033	0.715	1.492	0.865		
History of cerebrovascular disease	0.484	0.285	1.623	0.929	2.837	0.089		

GFRcre < 60 mL/min/1.73m^2

	Multivariate						Hosmer-Lemeshow test	
Independent variables	Coefficient†	SE	OR	OR [Lower]	OR [Upper]	P value	χ^2‡	P value §
Protein-bound uremic toxin score	0.324	0.093	1.383	1.153	1.659	<.001	5.488	0.704
Male gender	0.077	0.226	1.080	0.693	1.684	0.733		
Age	1.373	0.156	3.945	2.906	5.355	<.001		
Systolic blood pressure	-0.088	0.155	0.916	0.676	1.240	0.569		
Body mass index	0.293	0.134	1.340	1.030	1.743	0.029		
Diabetes mellitus	0.129	0.231	1.137	0.723	1.788	0.578		
History of cerebrovascular disease	1.120	0.360	3.066	1.515	6.207	0.002		

GFRcre; glomerular filtration rate estimated using standardized serum creatinine

‡: Chi-square value from the Hosmer-Lemeshow test; the lower the value, the better the fit.

§: P value from the Hosmer-Lemeshow test; the higher the value, the better the fit.

Table S14. Association of cognitive impairment, MMSE <23, with protein-bound uremic toxin score and clinical factors

	Univariate						Multivariate						Hosmer-Lemeshow test	
Independent variables	Coefficient†	SE	OR	OR [Lower]	OR [Upper]	P value	Coefficient†	SE	OR	OR [Lower]	OR [Upper]	P value	χ^2‡	P value §
Protein-bound uremic toxin score†	0.285	0.061	1.329	1.179	1.498	<.001	0.269	0.081	1.309	1.118	1.534	<.001	9.958	0.268
Male gender	0.285	0.147	1.330	0.996	1.775	0.053	0.263	0.169	1.301	0.934	1.813	0.120		
Age	1.334	0.114	3.796	3.034	4.750	<.001	1.346	0.123	3.843	3.021	4.889	<.001		
Systolic blood pressure	0.061	0.064	1.063	0.939	1.204	0.333	-0.023	0.106	0.978	0.795	1.202	0.831		
Body mass index	-0.217	0.080	0.805	0.689	0.941	0.006	0.077	0.098	1.080	0.891	1.310	0.430		
Diabetes mellitus	-0.279	0.159	0.757	0.554	1.033	0.079	0.201	0.182	1.223	0.856	1.748	0.269		
History of cerebrovascular disease	0.952	0.191	2.592	1.781	3.772	<.001	0.526	0.217	1.692	1.107	2.586	0.015		

†: Standardized coefficient; the higher the value, the greater the influence.

‡: Chi-square value from the Hosmer-Lemeshow test; the lower the value, the better the fit.

§: P value from the Hosmer-Lemeshow test; the higher the value, the better the fit.

Table S15. Association of continuous MMSE scores with protein-bound uremic toxin score and clinical factors

Independent variables	Coefficient	SE	Lower	Upper	P value
Protein-bound uremic toxin score	-0.126	0.0322	-0.189	-0.063	<.001
Female gender	0.112	0.0499	0.015	0.210	0.024
Male gender	0	.	.	.	.
Age	-0.471	0.0263	-0.522	-0.419	<.001
Systolic blood pressure	0.030	0.0219	-0.013	0.073	0.168
Body mass index	-0.029	0.0241	-0.076	0.019	0.233
Diabetes mellitus (-)	-9.78E-05	0.0493	-0.097	0.097	0.998
Diabetes mellitus (+)	0	.	.	.	.
History of cerebrovascular disease (-)	0.294	0.0826	0.132	0.456	<.001
History of cerebrovascular disease (+)	0	.	.	.	.

Figure S1: Protein-bound uremic toxins according to kidney dysfunction severity. Serum levels of PBUTs, including (A) indoxyl sulfate (IS), (B) p-cresyl sulfate (PCS), (C) indole acetic acid (IAA), (D) phenyl sulfate (PhS), and (E) hippuric acid (HA), are shown across CKD G categories: G1 (GFRcre ≥90 mL/min/1.73 m^2), G2 (GFRcre 60–89 mL/min/1.73 m^2), G3a (GFRcre 45–59 mL/min/1.73 m^2), G3b (GFRcre 30–44 mL/min/1.73 m^2), G4 (GFRcre 15–29 mL/min/1.73 m^2), and G5 (GFRcre <15 mL/min/1.73 m^2). GFRcre, glomerular filtration rate estimated using standardized serum creatinine. Data are shown as mean ± standard deviation (error bars).

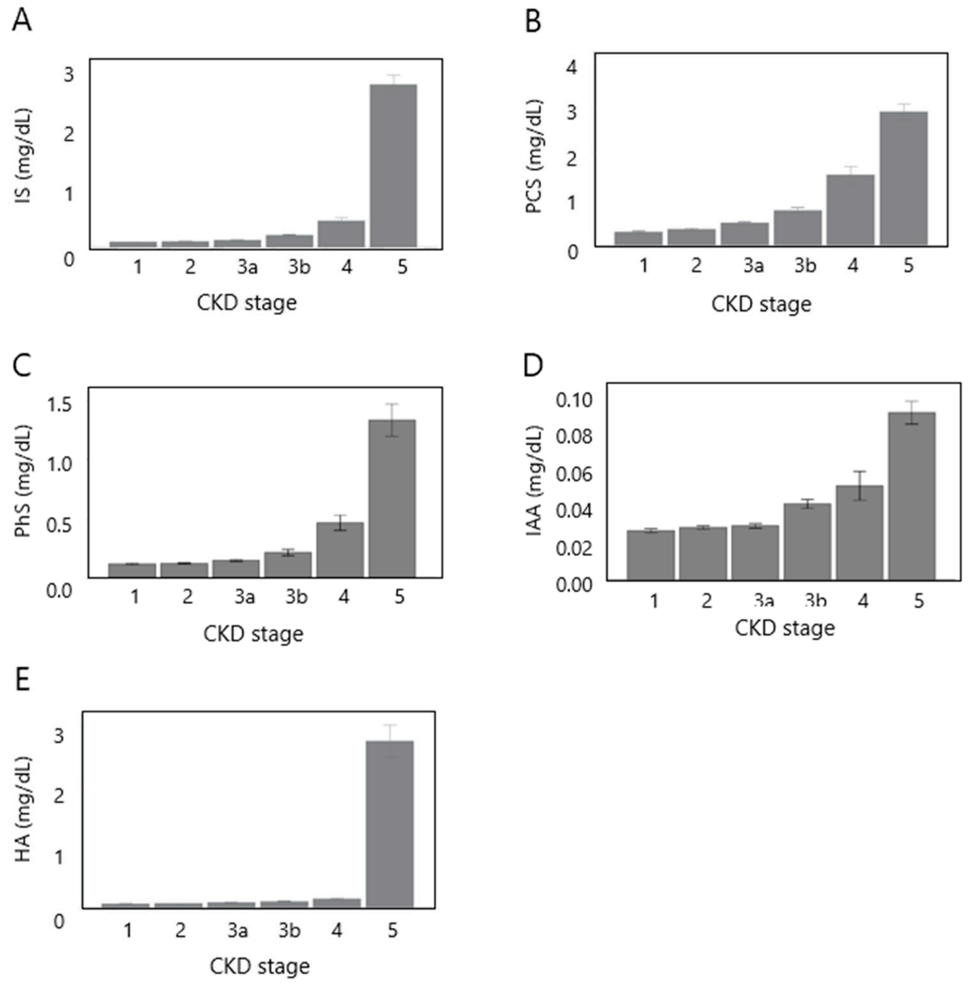

Figure S2. Scree plot for principal component analysis of protein-bound uremic toxins

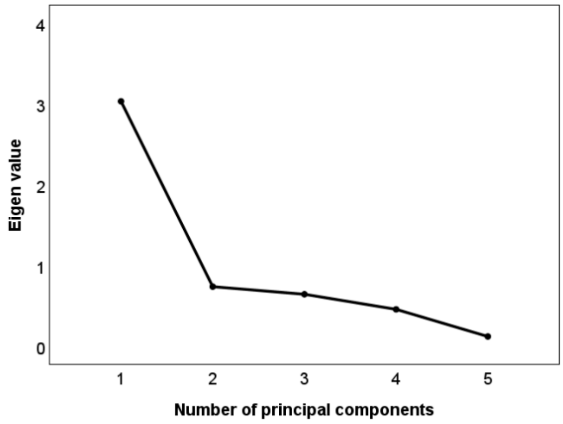

Figure S3. Flow diagram of participant selection

GFRcre, glomerular filtration rate estimated using standardized serum creatinine; GFRcys, glomerular filtration rate estimated using standardized serum cystatin C; PBUT, protein-bound uremic toxin; PROST, PROject in Sado for Total Health.

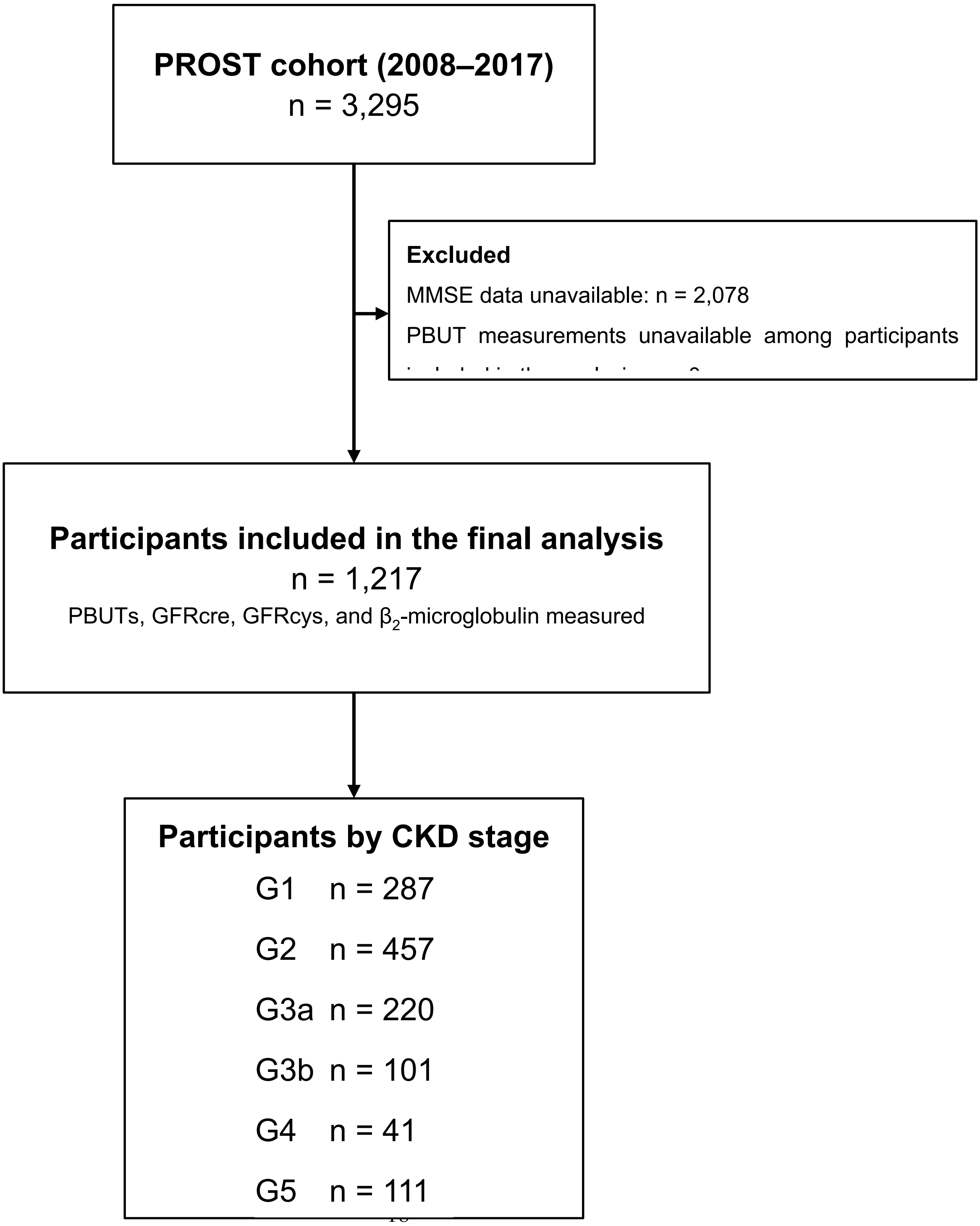

Supplement: Supplementary file 1 [file toxins-18-00422-s001.zip › toxins-4518717-supplementary.pdf]
